# Hybrid films loaded with 5-fluorouracil and Reglan for synergistic treatment of colon cancer via asynchronous dual-drug delivery

**DOI:** 10.3389/fbioe.2024.1398730

**Published:** 2024-06-13

**Authors:** Hairong Mao, Jianfeng Zhou, Liang Yan, Shuping Zhang, Deng-Guang Yu

**Affiliations:** ^1^ College of Chemistry and Chemical Engineering, Zhengzhou Normal University, Zhengzhou, Henan, China; ^2^ School of Materials and Chemistry, University of Shanghai for Science and Technology, Shanghai, China; ^3^ School of Optical-Electrical and Computer Engineering, University of Shanghai for Science and Technology, Shanghai, China

**Keywords:** synergistic therapy, hybrid films, colon cancer, asynchronous dual-drug delivery, coaxial electrospraying, casting, tumor-targeted therapy

## Abstract

Combination therapy with oral administration of several active ingredients is a popular clinical treatment for cancer. However, the traditional method has poor convenience, less safety, and low efficiency for patients. The combination of traditional pharmaceutical techniques and advanced material conversion methods can provide new solutions to this issue. In this research, a new kind of hybrid film was created via coaxial electrospraying, followed by a casting process. The films were composed of Reglan and 5-fluorouracil (5-FU)-loaded cellulose acetate (CA) core-shell particles in a polyvinylpyrrolidone (PVP) film matrix. Microscopic observations of these films demonstrated a solid cross section loaded with core-shell particles. X-ray diffraction and Fourier-transform infrared tests verified that the Reglan and 5-FU loaded in the films showed amorphous states and fine compatibilities with the polymeric matrices, i.e., PVP and CA, respectively. *In vitro* dissolution tests indicated that the films were able to provide the desired asynchronous dual-drug delivery, fast release of Reglan, and sustained release of 5-FU. The controlled release mechanisms were shown to be an erosion mechanism for Reglan and a typical Fickian diffusion mechanism for 5-FU. The protocols reported herein pioneer a new approach for fabricating biomaterials loaded with multiple drugs, each with its own controlled release behavior, for synergistic cancer treatment.

## 1 Introduction

Combined therapy for cancer is a popular clinical approach (Shen et al., 2020; Wu et al., 2022; Liu et al., 2023; Wang et al., 2024), wherein patients are frequently administered several kinds of dosage forms of the drugs for achieving synergistic anticancer effects in the clinic; this approach has low compliance owing to administration inconvenience (Verma et al., 2023; Tian et al., 2024). Furthermore, the side effects are often of significant concern (Huang et al., 2024a; Zhang et al., 2024a; Zhan and Zhang, 2024). One clinical practice of combined therapy entails oral administration of the therapeutics to the patients along with an initial antiemetic drug to prevent strong gastrointestinal discomfort; the main therapeutic drugs are administered approximately half an hour after the initial drug (Lang et al., 2023; Zhang et al., 2023). Thus, all-in-one dosage forms are always welcomed by patients to achieve safe, effective, and convenient drug delivery (Zhao et al., 2023). However, the fabrication of all-in-one dosage forms poses a significant challenge to researchers in the fields of pharmaceutics, materials science, engineering, nanoscience, and nanotechnology (Tan et al., 2023; Yang et al., 2023).

Compared to traditional pharmaceutical techniques such as tablets and pellets, pharmaceutical nanotechniques offer a series of advantages in tailoring the components, compositions, and spatial distributions when loading the active ingredients (Cai et al., 2022; Wang et al., 2023a; Cai et al., 2023; Mushtaq et al., 2023; Hao et al., 2024), and are therefore more powerful for creating novel all-in-one dosage forms or multifunctional medicated nanomaterials. One example of this is electrospinning, which is facile for loading various kinds of components within a nanofiber, provided they can dissolve in a certain solvent or solvent mixture (Kang et al., 2020; Yang et al., 2022; Peng et al., 2024). Alternatively, these components can be tailored to have their own chambers within the structured nanofibers, provided there are no co-dissolved solvents for blend electrospinning (Yao et al., 2018; Yao et al., 2022; Brimo et al., 2023). Moreover, such multichamber structured nanofibers can be produced in a single step in a straightforward manner (Yu et al., 2024). The nozzle of a spinneret at the macroscale can be explored as a template to duplicate a wide variety of complex multicompartment nanoproducts through electrospinning (Song et al., 2023; Tabakoglu et al., 2023). These fundamental multichamber nanoproducts include but are not limited to, the core-shell (Shi et al., 2024a; Han et al., 2024), Janus (Zhang et al., 2024b; Yan et al., 2024; Zhou et al., 2024b), trilayer core-shell (Wang et al., 2020; Wang et al., 2023b), trisection Janus (Wang et al., 2017; Jiang et al., 2024; Xu et al., 2024b), combinations of Janus and core-shell (Li et al., 2024), and abundant derivatives (Li et al., 2022; Guler et al., 2023) of these multichamber structures. For example, the derivatives of trilayer core-shell nanofibers can be used as drug gradient distribution nanofibers for sustained release (Wang et al., 2023c) and as drug discrete distribution nanofibers for accurate biphasic release (Liu et al., 2024). Among these multichamber structures, the double-layer core-shell structure is the most fundamental form (Qain et al., 2023; Chen et al., 2024a), which has received abundant attention in almost all scientific fields (Cao and Ding, 2022; Shi et al., 2024a). Electrospun core-shell nanofibers have been widely explored for potential applications in tissue engineering (Wang et al., 2017; Zhu et al., 2024), food packaging (Huang et al., 2022), energy conversion and storage (Yu et al., 2023; Wei et al., 2024), wound dressing (Khan et al., 2024), functional fabrics (Zhang et al., 2024c), drug delivery (Xie et al., 2022; Gong et al., 2024), and treatment of pollutants in air, water, and soil (Song et al., 2022; Li et al., 2023; Su et al., 2024). It is therefore clear that these multichambered structures, particularly the core-shell structures, play important roles in the development of novel all-in-one dosage forms for cancer therapy.

Compared with electrospun core-shell nanofibers, electrosprayed core-shell micro, and nano particles have received less attention, even though both are top-down nanofabrication methods having obvious advantages over bottom-up methods during large-scale production (Isaacofft and Brown, 2017; Ahmed et al., 2024; Chen et al., 2024b). A simple search in Web of Science returns 1,421 items with “electrospun core-shell nanofibers” or “electrospun core-sheath nanofibers” as the search topic, whereas the number of items returned for “electrosprayed core-shell particles” or “electrosprayed core-shell microparticles” or “electrosprayed core-shell nanoparticles” is only 59 (search date: 2024-Jan-27). This finding is attributed to the fact that the core-shell medicated particles are often reported to be widely prepared using bottom-up chemical synthesis methods, which make it difficult for creating core-sheath nanofibers. In addition to the advantages similar to those of coaxial electrospinning, such as the single-step fabrication, straightforward implementation, and manufacture using structured nozzles of the spraying heads as templates, the coaxial electrospraying approach for creating core-shell particles has the advantage of being able to treat a broader range of raw materials through top-down fabrication (Ji et al., 2023; Xu et al., 2023). There are only approximately 200 filament-forming polymers that can be converted to nanofibers through electrospinning, and these often have narrow electrospinnable windows (Sivan et al., 2022a; Yu et al., 2024). In sharp contrast, there are numerous materials that can be transformed into particles via electrospraying (Sun et al., 2023). Furthermore, electrospraying enables the preparation of solid particles from dilute polymeric solutions (Tabakoglu et al., 2023), and this process can be scaled up more easily than many of the bottom-up particulate synthesis processes.

Although new nanotechniques have become popular for creating novel functional nanomaterials (Wang and Feng, 2022; Wu and Li, 2022; Dong et al., 2023), real applications of nanobiomaterials in the clinic and for commercial purposes remain very limited (Xuan et al., 2023; Huang et al., 2024b; Yu and Zhou, 2024). There are still numerous dosage forms available in drugstores that are prepared using traditional pharmaceutical techniques and could one day be replaced by advanced nanoproducts. Accordingly, based on expanding the real applications of nanodrug-delivery systems (DDSs) prepared using advanced nanotechniques, it is hypothesized that combinations of advanced nanomethods with traditional pharmaceutical techniques would enable new methods for producing novel hybrid DDSs containing nanoscale medicated materials. Based on this proof-of-concept idea, this study investigates the combination of coaxial electrospraying and traditional film casting to produce novel anticancer DDSs. The all-in-one casting films contain both electrosprayed core-shell particles loaded with 5-fluorouracil (5-FU, sustained release) and Reglan (rapid release) in a homogeneous distribution prepared by a combination technique, which is anticipated to provide asynchronous dual-drug delivery for synergistic treatment of colon cancer.

The desired asynchronous dual-drug delivery approach comprises a first-stage release of Reglan, which was realized through fast dissolution of the soluble matrix, i.e., polyvinylpyrrolidone K30 (PVP K30), under acidic conditions. The second-stage release of 5-FU was achieved using electrosprayed core-shell cellulose acetate (CA) nanoparticles under medium conditions that were freed by the dissolution of the casting films in the first stage. Reglan is designed to be released in a pulsatile manner, which is favored by patients owing to its therapeutic effect of preventing vomiting. Reglan is also called metoclopramide or chloramphenicol; it is a white to light-yellow crystalline powder with the molecular formula C_14_H_22_ClN_3_O_2_. It is also known as 4-amino-5-chroro N-(2-diethylamine)-2-methoxybenzamide. Reglan is soluble in chloroform, slightly soluble in ethanol or acetone, and almost insoluble in ether and water, but soluble in an acidic solution. Reglan has a strong central antiemetic effect and is frequently exploited for nausea, vomiting, belching, loss of appetite, and indigestion (Shafi et al., 2023). The drug 5-FU is a white crystalline powder that is slightly soluble in water and ethanol, almost insoluble in chloroform, and soluble in dilute hydrochloric acid or sodium hydroxide solution. It is an antimetabolic and antitumor drug whose local concentration maintenance time is positively correlated with the therapeutic effect. Its plasma half-life is extremely short (10–30 min), thus requiring frequent administration; it also has significant side effects, because of which sustained release is highly desired by patients (Xie et al., 2018; Feng et al., 2022).

## 2 Materials and methods

### 2.1 Materials

The drugs 5-FU and Reglan were purchased from TCI Shanghai Co., Ltd. (Shanghai, China). The polymeric matrices PVP K30 (*M*_w_ = 58,000) and CA (*M*_w_ = 50,000) were purchased from BASF Shanghai Co., Ltd. (Shanghai, China) and Shanghai Haosheng Biotechnol. Co., Ltd. (Shanghai, China), respectively. The organic solvents anhydrous ethanol, chloroform (analytical grade), and dimethylacetamide (DMAc) were obtained from Sinopharm Reagent Co., Ltd. (Shanghai, China). All the other raw materials were of analytical grade, and water was double-distilled before use.

### 2.2 Combined process of fabricating all-in-one medicated films

After some preliminary experiments, four working fluids were determined for the combined fabrication process: 1) For preparing the monolithic 5-FU/CA composite particles, 3.0 g of 5-FU and 9.0 g of CA were co-dissolved in 300 mL of a solution containing DMAc, acetone, and ethanol in a volume ratio of 2:3:1 correspondingly. 2) To prepare the core-shell particles via coaxial electrospraying, the core fluid was prepared by dissolving 6.0 g of 5-FU and 9.0 g of CA in 300 mL of a solution containing DMAc, acetone, and ethanol in a volume ratio of 2:3:1; further, the shell fluid was prepared by dissolving 9.0 g of CA in 300 mL of a solution containing DMAc, acetone, and ethanol in a volume ratio of 1:4:1. 3) For the casting film preparation, 3.0 g of Reglan and 15.0 g of PVP K30 were dissolved in 100 mL of chloroform, and 12.0 g of 5-FU-loaded core-shell particles were suspended in this Reglan/PVP solution.

A homemade concentric spinneret was used to set up the coaxial electrospraying apparatus. The other parts included two fluid drivers (KDS100 and KDS200, Cole-Parmer, United States of America) for quantitatively pumping the core and shell working fluids with an accuracy of 0.01 mL/h, a high-voltage generator (60 kV/2 mA, Wuhan Hua-Tian High Power Co., Ltd., Wuhan, China), and a collector comprising a cardboard wrapped with aluminum foil. After some optimizations, the working conditions were determined as follows: core and shell fluid flowrates of 1.0 and 1.0 mL/h, respectively; an applied voltage of 18 kV; and a collection distance of 20 cm between the nozzle of the spinneret and collector. The suspensions containing Reglan and core-shell particles were degassed through an ultrasonic instrument in an ice bath for better implementation (Wang et al., 2023a). Later, the suspensions were placed in an oven at a temperature of 40°C until a constant weight was achieved. Other parameters are included in Table 1.

**TABLE 1:** Parameters for manufacturing the electrosprayed medicated products.

No.	Production	Parameters for electrospraying^a^				Products	Drug content (%)	
		*V* (kV)	*F* (mL/h)		*D* (cm)			
			Core	Shell			Reglan	5-FU
S1	Single-fluid blend electrospraying	18	--	2.0	20	Dented concave particles	--	--
S2	Single-fluid blend electrospraying	18	2.0	--	20	Spherical particles	--	25%
S3	Coaxial electrospraying	18	1.0	1.0	20	Core-shell particles	--	25%
S4	Coaxial electrospraying and film casting	18	1.0	1.0	20	Solid films	10%	10%

^a^ The symbols *V*, *F,* and *D* represent the applied voltage, fluid flow rate, and distance between the nozzle of the spinneret and collector, respectively.

### 2.3 Characterizations

#### 2.3.1 Morphology and inner structure

The surface morphologies of the Electrohydrodynamic atomization (EHDA) products (S1, S2, and S3) were assessed by scanning electron microscopy (SEM, FEI Quanta G450 FEG, Inc., Hillsboro, OR, United States of America). To render their electrical conductivities, the samples were sputter-coated with gold in an argon atmosphere before evaluation, and the images were obtained at an excitation voltage of 10 keV. The inner structures of the electrosprayed particles S2 and S3 were evaluated by transmission electron microscopy (TEM, JEM2100F, JEOL, Tokyo, Japan). A lacy carbon-coated copper grid was fixed on the collector for approximately 2 min during the sampling processes. Then, cross sections of the hybrid films were obtained through direct manual breakage. The diameter and size distributions of the electrosprayed particles were evaluated based on measurements from over 100 locations on the SEM images.

#### 2.3.2 Physical state and compatibility

A Bruker D8 Advance X-ray diffractometer (XRD, Bruker, Bremen, Germany) was utilized to obtain the XRD patterns, which were recorded from 10° to 60° in the continuous mode at a scanning speed of 5°/min and step size of 0.02°. The attenuated total reflection Fourier transform infrared (ATR-FTIR) spectra were then recorded using a Spectrum 100 spectrometer (Perkin-Elmer, Waltham, MA, United States of America) in the scanning range of 500–4,000 cm^-1^ with a resolution of 2 cm^-1^.

### 2.4 Drug loading efficiency and *in vitro* dissolution tests

The drug loading efficiencies (LEs, %) were calculated according to Eq. (1):

$$LE\left(\%\right)=\frac{Q_{d}}{Q_{p}}\times 100\%$$ (1)

where *Q*_d_ and *Q*_p_ represent the detected and theoretical amounts of the drugs during preparation. To prepare the electrosprayed medicated particles S2 and S3, specific amounts of their powders were weighed and dissolved in a solution containing DMAc, acetone, and ethanol in a volume ratio of 2:3:1. Then, approximately 1 mL of each solution was dripped into 100 mL of water under ultrasonic conditions. After filtration, the aqueous solution was measured to determine the 5-FU loading in the particles. The *LE* (%) value of Reglan in the solid film was determined through an *in vitro* dissolution test.

The *in vitro* dissolution tests were conducted in accordance with the Chinese Pharmacopoeia (2020 Ed.), and the paddle method was employed along with an RCZ-8A dissolution apparatus (Tianjin University Radio Factory, China) and seven vessels. The test conditions involved a rotation speed of 50 rpm and a dissolution media temperature of 37°C ± 1°C. For the medicated products S2, S3, and S4, powders of respective weights of 0.1 g, 0.1 g, and 0.25 g were placed in the vessels with a constant 5-FU feed. The dissolution media (600 mL) used was 0.01 N HCl (pH = 2.0) for the first 2 h to mimic the gastric juices artificially, and an equivalent volume of sodium hydroxide was subsequently added to the dissolution media to adjust the pH to 7.0 to simulate the intestinal fluids artificially. For the S4 sample, the residues from the *in vitro* dissolution test were separated and dried naturally for SEM observations.

At predetermined time intervals, 5.0 mL volumes of the dissolution media were withdrawn for sampling, and equal volumes of fresh media were added to maintain constant volumes. The absorbance values of the samples were measured using a UV‒vis spectrophotometer (Unico Instrument Co., Ltd., Shanghai, China). The amounts of Reglan and 5-FU in the samples were then calculated using their predetermined calibration curves. No mutual interference was observed between the dissolutions of Reglan and 5-FU due to the blank CA coating in the core-shell particles.

### 2.5 Statistical analysis

All the experimental data are presented as mean ± SD. The results from the *in vitro* dissolution tests were analyzed using a one-way ANOVA, and the significance level threshold was set at 0.05. Thus, *p* (probability) values lower than 0.05 were considered to be statistically significant.

## 3 Results and discussion

### 3.1 Combined coaxial electrospraying and fluid casting

**FIGURE 1:**
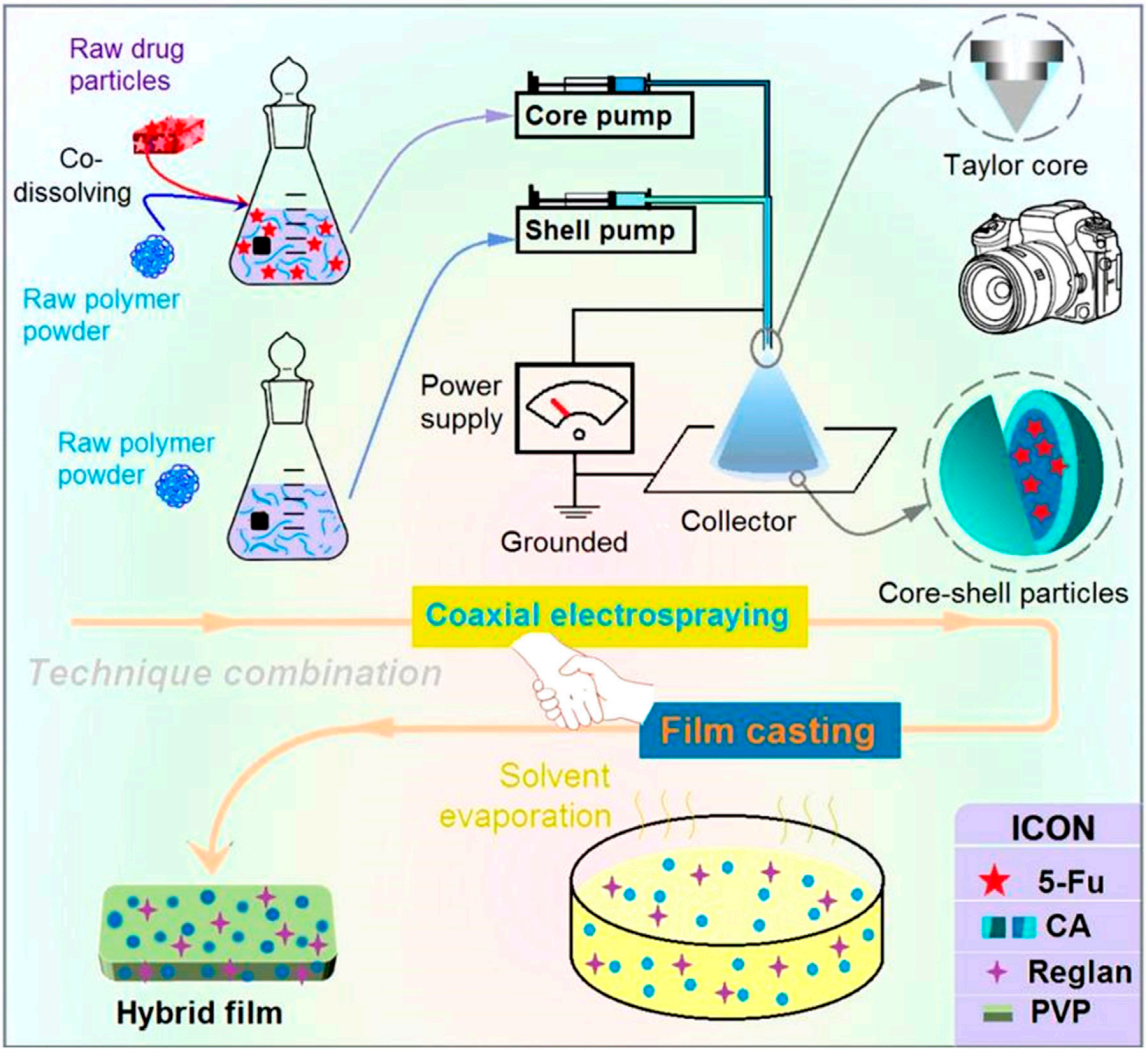
Diagram showing the combination of coaxial electrospraying and fluid casting techniques to create a hybrid medicated film loaded with two synergistic drugs.

Electrospinning as an advanced nanofabrication technique is rapidly gaining applications in a wide variety of fields (Bai et al., 2022; Cao et al., 2022; Chen et al., 2023a; Shi et al., 2024b); its success is attributable to a series of unique properties, such as straightforward, single-step, and flexible fabrication, in addition to the use of an inexpensive apparatus (Sivan et al., 2022b; Lv et al., 2024a; Lv et al., 2024b). Among these, its flexibility allows conveniently combining electrospinning with many traditional techniques and advanced chemical and physical methods (Yu and Xu, 2023; Yu and Zhou, 2024). Similar to electrospinning, electrospraying also allows facile combinations with other fabrication methods to develop new material conversion approaches. A diagram showing the compatibility between coaxial electrospraying and solvent casting is depicted in Figure 1.

Effective conversion of the electrosprayed medicated particles to suitable dosage forms is always an important process in drug delivery (Alfatama et al., 2024). After preparation, the core-shell particles can be placed in capsules for direct oral administration or subjected to additional processing to add new types of functions. Solvent casting is a common method for creating lipid and polymeric films in pharmaceuticals. The further casting of the electrosprayed core-shell particles is useful not only for convenient oral delivery and easy shipping and storage but also for loading new active ingredients for synergistic therapy. This proof-of-concept idea is expected to be useful for developing other multifunctional biomaterials in the future.

**FIGURE 2:**
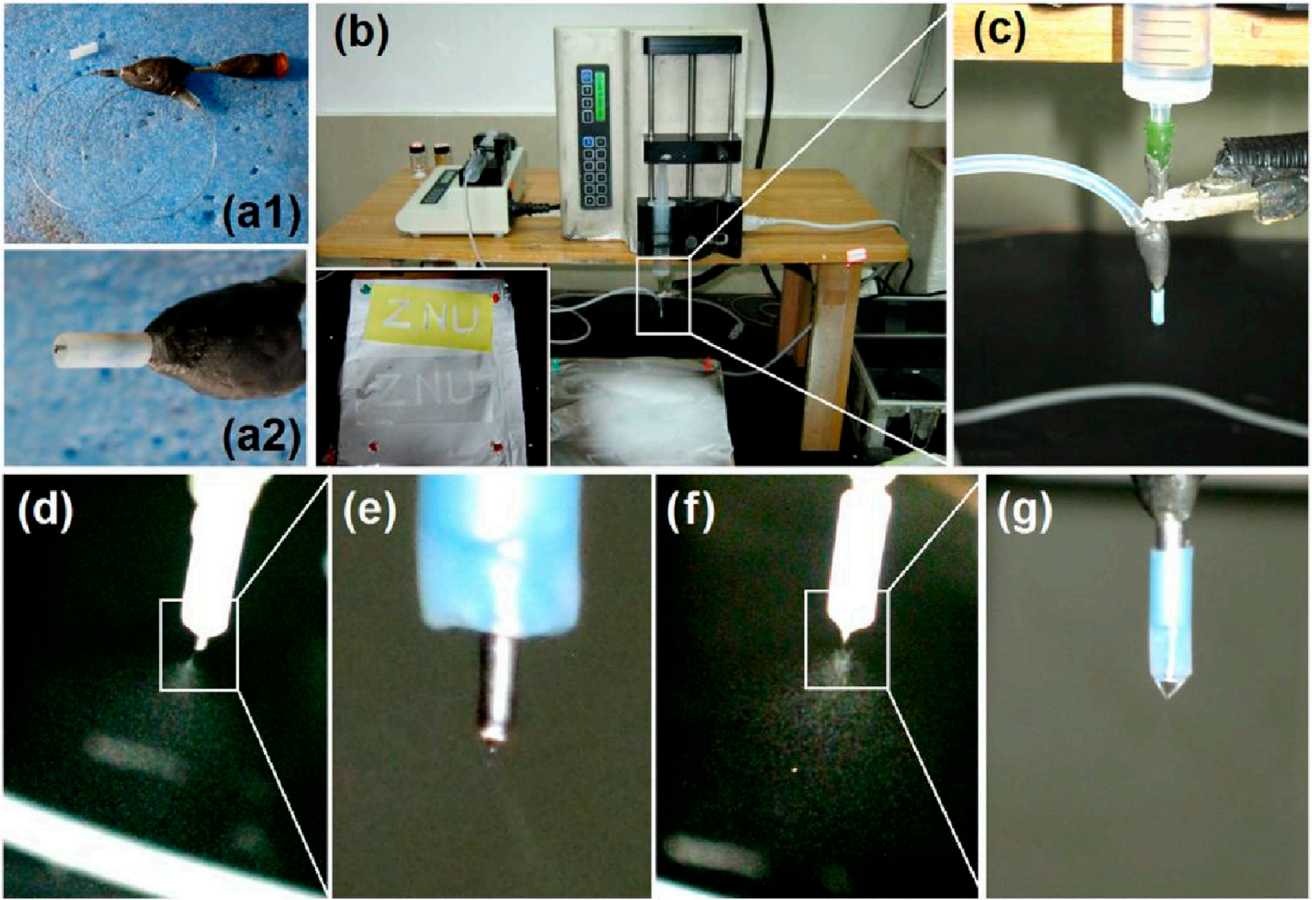
Implementation of coaxial electrospraying: **(a1)** and **(a2)** a detachable concentric spinneret as a key part of the coaxial electrospraying apparatus; **(B)** digital image of the coaxial electrospraying apparatus for creating the core-shell particles S3, where the bottom-left inset indicates the deposition of electrosprayed particles for three letters (ZNU); **(C)** connections of the spinneret with two working fluids and an alligator clip from the power supply; **(D)** typical electrostatic atomization process and **(E)** its Taylor cone for generating homogeneous CA-5-FU particles; **(F)** coaxial atomization process and **(G)** its typical compound Taylor cone.

It must be noted that coaxial electrospraying is the key technique in the proposed combination. In this study, a detachable spinneret was developed for the coaxial electrospraying process, which has also been used in other EHDA processes, such as single-fluid electrospinning and coaxial electrospinning (Chen et al., 2023b; Zheng et al., 2024). The Teflon-coated concentric spinneret consists of a common metal-based concentric spinneret and a set of Teflon tubings (Figure 2a1). The Teflon tubing can be moved vertically to adjust the nozzle surfaces of the inner and outer capillaries, which allows further adjustments of the behaviors of the shell and core working fluids (Figure 2a2).

The complete homemade electrospraying system is shown in Figure 2B and comprises two pumps, a collector, a spinneret, and a power supply (Alfatama et al., 2024). Under optimized experimental conditions, the typical process for generating the core-shell particles S3 is as shown in Figure 2B; the inset image on the bottom left shows the deposition of the electrosprayed particles over the letters “ZNU” formed by covering the surface of the collector with a sheet of paper for approximately 10 min. During the process, the area around the spinneret that is connected to the two working fluids is considered important, along with the alligator clip connected to the power supply (Figure 2C). All the electrospraying processes are initiated at this point of convergence upon the reasonable formation of a stable Taylor cone (Ji et al., 2023; Xu et al., 2023).

To prepare the homogeneous 5-FU/CA composite particles S2, only the core metal capillary was used to guide the working fluid toward the nozzle of the spinneret. After applying a high voltage of 18 kV, the atomization can be discerned, as shown in Figure 2D. The atomization process consisted of three successive steps, i.e., Taylor cone (Figure 2E) formation, a straight fluid jet (often condensed to a point at the tip of the Taylor cone), and Coulombic explosion region formation. When coaxial electrospraying was performed, the Teflon tubing was moved downward to allow outward projection of only 0.2 mm of the core metal capillary. This arrangement was favorable for fine encapsulation of the core fluid by the shell fluid. The atomization is recorded in Figure 2F. The working process here is apparently similar to the single-fluid process comprising the Taylor cone, joint point, and successive Coulombic explosion sections. However, the different compounds of the Taylor cone can be discerned by further enlarging the observations, as shown in Figure 2G.

### 3.2 Morphologies and inner structures of electrosprayed particles and hybrid films

**FIGURE 3:**
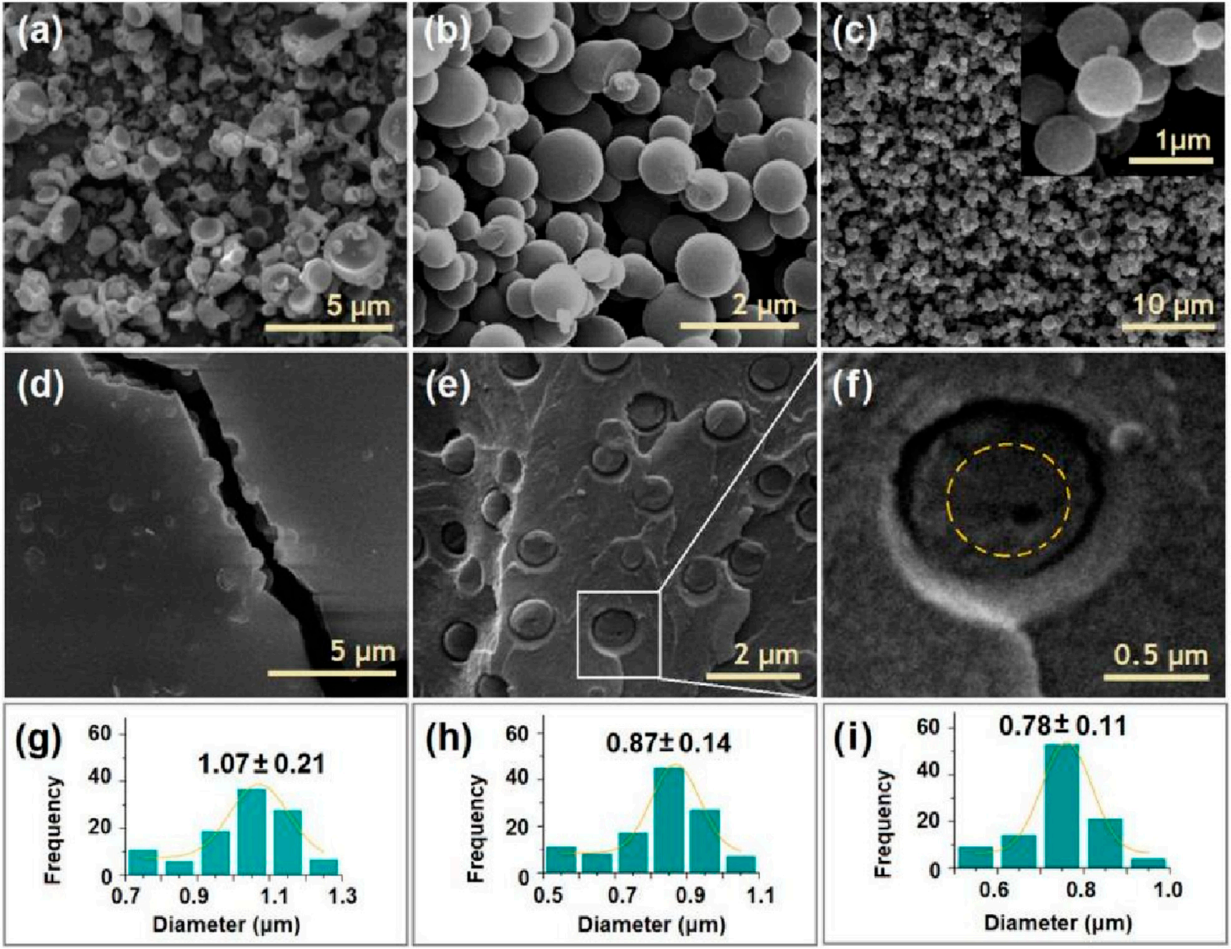
Scanning electron microscopy (SEM) images of the prepared particles and their diameter distributions: images of the **(A)** blank CA particles S1, **(B)** homogeneous 5-FU/CA particles S2, and **(C)** core-shell particles S3. **(D)** Surface morphology of the casting film via fission, **(E)** image of the cross-section of the casting film, and **(F)** image of a complete independent particle. **(G–I)** Diameter distributions of the electrosprayed particles S1, S2, and S3, respectively.

The morphologies and inner structures of the electrosprayed particles and hybrid films are shown in Figure 3. As anticipated, the CA particles S1 electrosprayed from the blank polymeric solution had a somewhat concave morphology, as shown in Figure 3A. Meanwhile, there are many satellites around the electrosprayed particles. In contrast, the 5-FU/CA particles S2 had a spherical morphology, as indicated in Figure 3B. Similarly, the core-shell particles S3 from the coaxial electrospraying process had round shapes, as exhibited in Figure 3C and in the inset image on the top right at a magnification of ×50,000. In general, the hybrid films were brittle, and cracks were observed after being manually broken by external forces, as shown in Figure 3D. The SEM morphologies of the hybrid films are shown in Figures 3E, F at various magnifications. From Figure 3F, it is clear that the surrounding regions are rougher than the central sections of the particles, indicating that each particle had a double-compartment core-shell structure.

Electrosprayed particles often have a broader diameter distribution than electrospun nanofibers; the particle sizes and morphologies are influenced by a series of solution properties and operational parameters. In this study, pure CA particles S1 (Figure 3G), 5-FU/CA composite particles S2 (Figure 3H), and core-shell particles S3 (Figure 3I) had average diameters of 1.07 ± 0.21 μm, 0.87 ± 0.14 μm, and 0.78 ± 0.11 μm, respectively. It is interesting to note that the composite particles S2 and the core-shell particles S3 had smaller diameters and more uniform size distributions than the blank CA particles S1, even though high drug loading was performed within S2 and S3.

**FIGURE 4:**
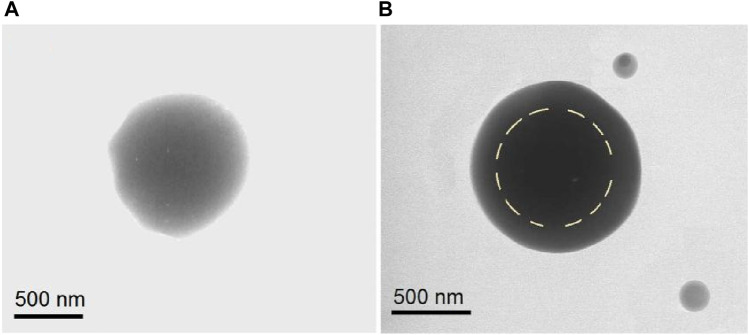
Transmission electron microscopy (TEM) images of the **(A)** homogeneous 5-FU/CA particle S2 from single-fluid blend electrospraying and **(B)** core-shell particle S3 from coaxial electrospraying.

TEM images were obtained to evaluate the inner structures of the composite particles S2 and core-shell particles S3. As seen in Figure 4A, the 5-FU/CA particles S2 had homogeneous structures. Under a bright field, the gray levels of the TEM images are a result of thickness, density, and elements. The gradually decreasing gray levels in Figure 4A are attributed to the thicknesses of the particles, with the darkest level at the center and the lightest levels near the boundaries. Overall, the elements and densities were similar throughout the particles. In contrast, the core-shell particles S3 had stepwise gray level changes, as indicated by the dashed circular line in Figure 4B. The reasons for these stepwise changes include different elements (the shell had no 5-FU), different thicknesses, and different densities.

**FIGURE 5:**
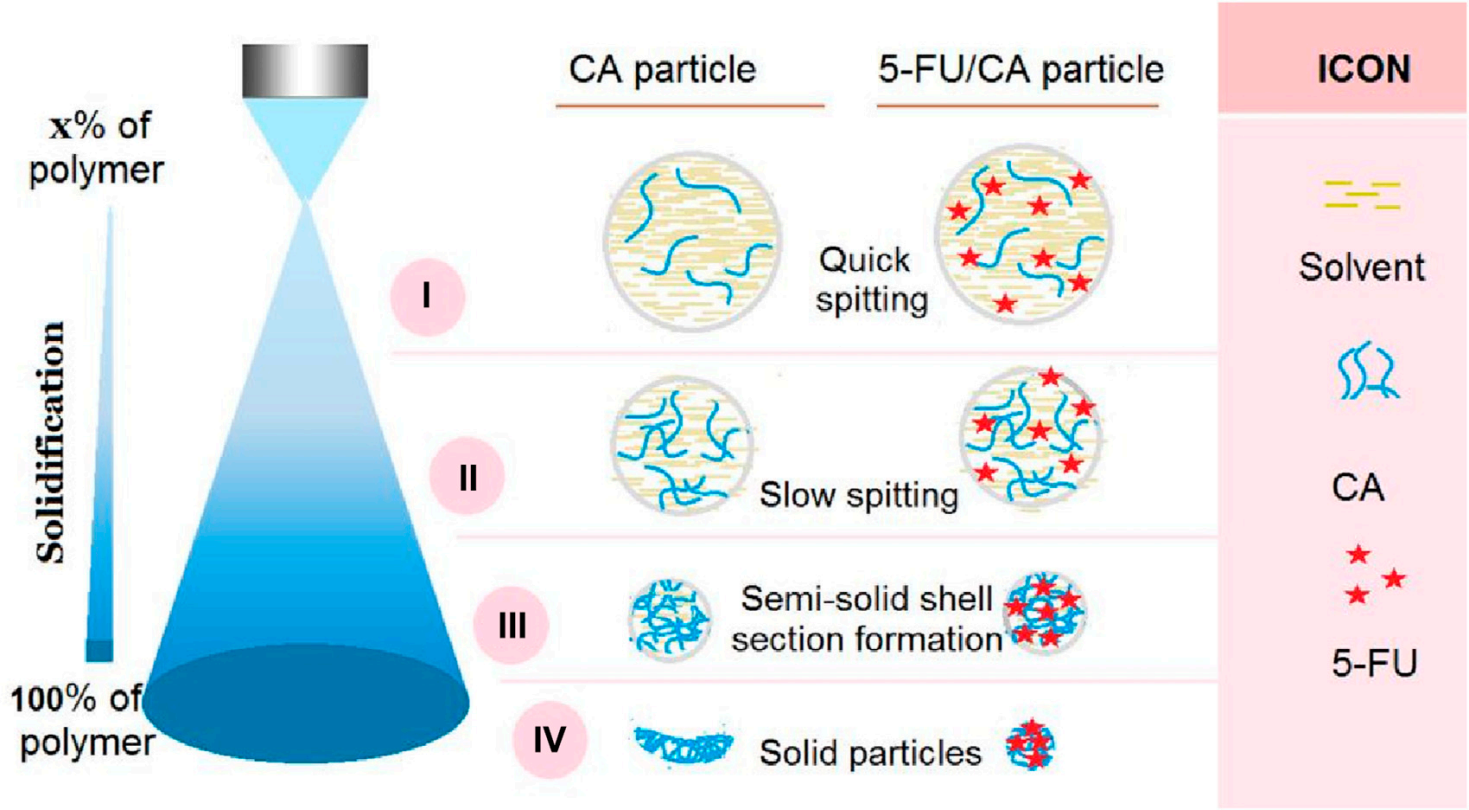
Suggested mechanisms for the microformation of concave particles S1 from electrospraying of pure CA solution and the formation of relatively spherical particles S2 from electrospraying of CA and 5-FU blend solution. The four steps of the mechanism are: i) quick splitting, ii) slow splitting, iii) semisolid shell formation, and iv) particle solidification.

The microformation mechanisms of the blank CA particles S1 and composite 5-FU/CA particles are compared in Figure 5. In general, the solidification processes of electrospinning and electrospraying are very different; the former is mainly a fluid drawing process, whereas the latter is a continuous fission process. From the Taylor cone to the collector, the working fluid experiences numerous fission reactions; each fission reaction inevitably results in a downsizing of the fluid droplets, a decrease in the solvent amount and related increases in the polymer concentrations within the droplets, a weakening of the surface charges and splitting forces, and proximity to the collector. The fission reactions continue until the repelling forces cannot split the droplets/solid particles/semisolid particles further.

Thus, we divide the entire Coulombic process into four subprocesses, as indicated in Figure 5. These four steps are: i) quick splitting; ii) slow splitting; iii) semisolid shell formation; and iv) particle solidification. The most common characteristics of each step are as follows. The quick splitting occurs after Taylor core formation when the strongest repelling forces atomize the fluid with the lowest solute concentration. Next, the slow splitting continues with the fluid droplets that have smaller repelling forces but high solute concentrations. Here, the surface and inner sections still have homogeneous components and compositions. When the droplets further approach the collector, a semisolid surface forms on the split droplet, whose surface and inner sections have heterogeneous components and compositions. Finally, the solidified particles from the semisolid surface droplets may still contain some solvents in their inner sections; owing to the presence of these residual solvents, the solidified particles may experience a barometric pressure and are flattened to concave morphologies. This is the mechanism of formation of the blank CA particles S1.

However, the addition of a large amount of 5-FU to the CA fluid can exert a remarkable influence on the entire electrospraying process. First, these small molecules may retard the formation of semisolid substances and compact membranes on the surfaces of the split droplets, thereby facilitating the fast removal of the solvent molecules from the inner sections of droplets. Second, when the collected particles experience a barometric pressure owing to the evaporation of the residual solvent molecules, the loaded molecules act as supports between the macromolecular chains to prevent deformation. This is the mechanism of formation of the spherical 5-FU/CA composite particles S2. As for the core-shell particles S3, the diluted shell CA solution may further facilitate the removal of the inner residual solvent molecules, thereby further guaranteeing a spherical morphology.

### 3.3 Physical states of the components and their compatibility

**FIGURE 6:**
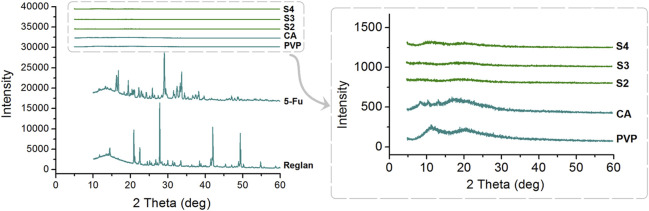
X-ray diffraction (XRD) patterns of the four starting components (Reglan, 5-FU, PVP, and CA) and their homogeneous composite S2, heterogeneous composite S3, and hybrid casting film S4.

For drug delivery, particularly for drugs with poor water solubilities, the amorphous state is more favorable than the crystalline state for predictable, controlled release (Pattnaik et al., 2023; Sun et al., 2024). The XRD patterns of the four starting components (Reglan, 5-FU, PVP, and CA) and their homogeneous composite S2, heterogeneous composite S3, and hybrid casting films S4 are shown in Figure 6. As indicated by the abundant sharp Bragg peaks in the patterns, both the raw 5-FU and Reglan powders are crystalline components, whereas the polymeric matrices CA and PVP are amorphous substances. When the working fluids are processed through single-fluid electrospraying, coaxial electrospraying, and solvent casting, all the converted solid products, i.e., monoaxial particles S2, core-shell particles S3, and hybrid films S4, are obtained in the desired amorphous state. The reasons for this are mainly attributed to the drug distribution on the molecular scale within the polymeric matrices, which resulted from the homogeneous working fluids, the extremely fast drying effect of electrospraying, and the fine compatibility between the drug and polymer molecules.

**FIGURE 7:**
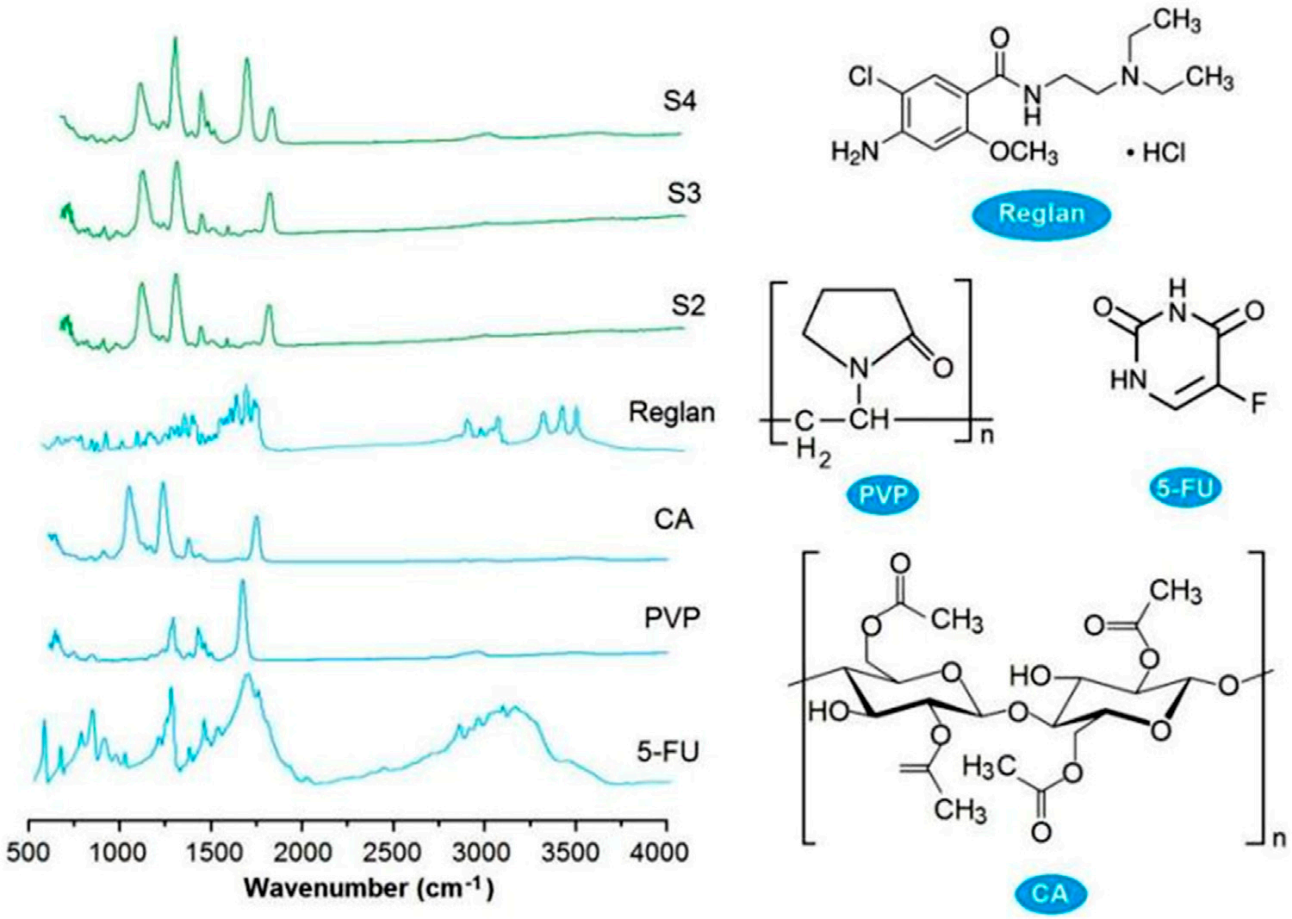
ATR-FTIR spectra of the four starting components (Reglan, 5-FU, PVP, and CA on the left) and their molecular formulas (right).

Good compatibility between a drug and its carrier is important for realizing not only the desired controlled release profile of the drug but also chemical and physical stability for storage and shipping. New types of polymers are continuously being tested for potential biomedical applications (Guo et al., 2021; Köse et al., 2022; Liao et al., 2023), and their compatibility with the loaded drugs is being disclosed (Ejeta et al., 2022; Jurić et al., 2023; Langer and Peppas, 2024; Song et al., 2024). FTIR spectrometry is one of the popular methods used to assess the compatibilities of components. In this study, the ATR-FTIR spectra of the four starting components, i.e., Reglan, 5-FU, PVP, and CA (left), and their molecular formulas (right), are shown in Figure 7. Comparing the spectra of the drugs with those of the loaded products, it is obvious that there are many observable sharp peaks for the raw drug powders; however, all of these were remarkably reduced or even attenuated in the spectra of the final products. This phenomenon suggests that the drug molecules interact with the polymeric molecules through secondary physical interactions, such as hydrogen bonding, hydrophobic interactions, or electrostatic interactions. Although these interactions cannot be directly detected using instruments, they can be deduced through their molecular formulas.

### 3.4 Asynchronous dual-drug delivery performance

The *LE* (%) values for 5-FU in the electrosprayed particles S2 and S3 were 99.26% ± 2.14% and 100.3% ± 1.77%, respectively. The electrospraying processes, regardless of the single-fluid blend process for creating homogeneous S2 or the coaxial process for producing core-shell S3, are essentially physical drying processes that are completed very rapidly. Thus, the drug 5-FU, without sublimation or volatilization properties, can be completely encapsulated in the solid particles.

**FIGURE 8:**
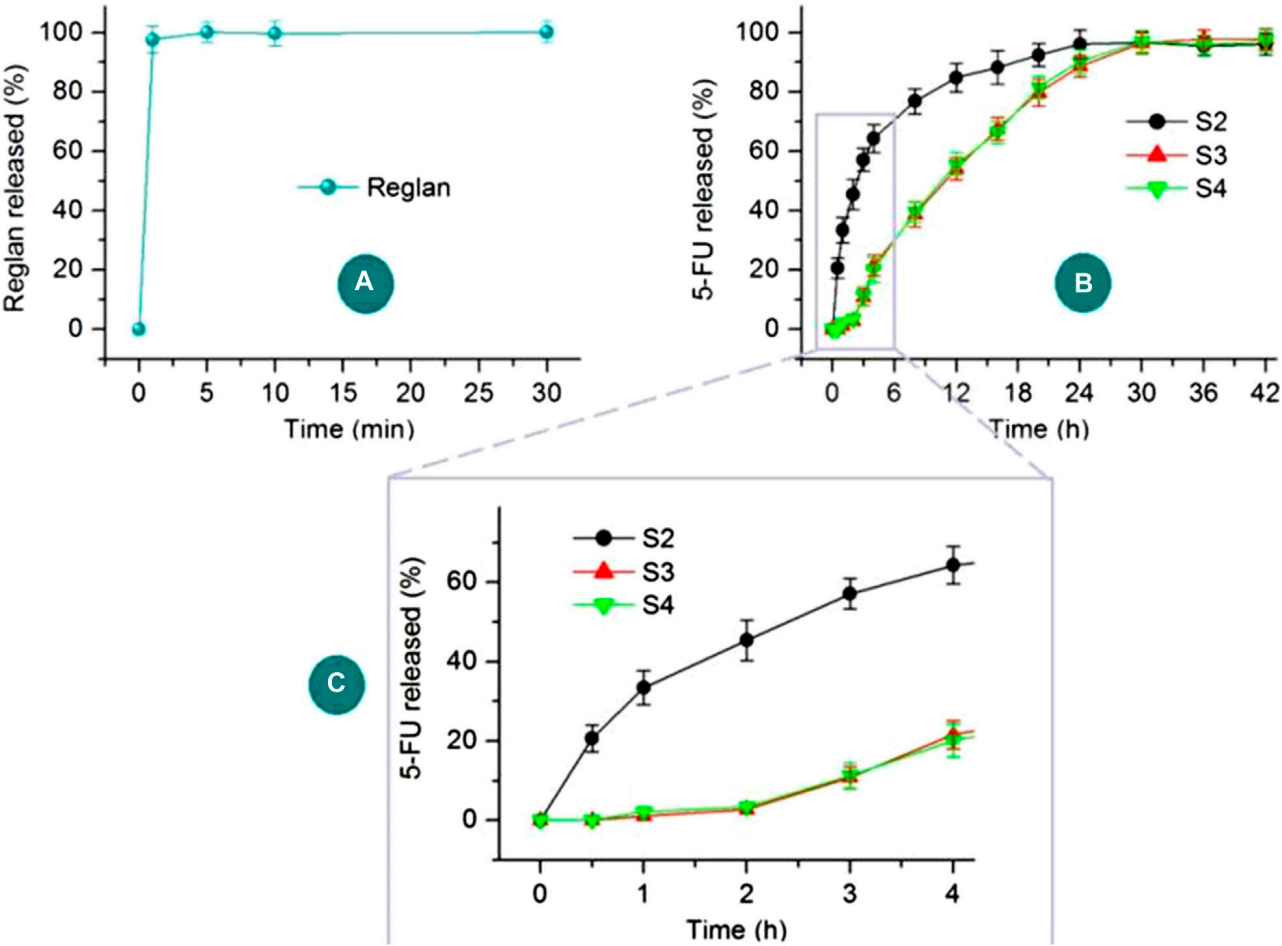
*In vitro* drug release profiles of Reglan and 5-FU from their host polymeric matrices: **(A)** Reglan release from the PVP matrix of the hybrid film; **(B)** 5-FU release profiles from S2, S3, and S4 over the entire experimental duration; **(C)** 5-FU release profiles from S2, S3, and S4 during the initial 4 h.

The *in vitro* drug release profiles of Reglan and 5-FU from their host polymeric matrices are shown in Figure 8. The antiemetic drug Reglan is released rapidly when the hybrid films are placed in the dissolution media, as indicated in Figure 8A. Meanwhile, the detected total release content of Reglan reached 99.47% ± 3.57% of the theoretical content calculated from the film casting suspensions. The pulsatile release of Reglan is highly desirable for patients because it allows for rapid initiation of antiemetic actions. As for 5-FU release from the corresponding products, i.e., 5-FU/CA composite particles S2, core-shell medicated particles S3, and particles released from the hybrid films S4, the profiles are compared in Figure 8B. The 5-FU release profiles over the initial 4 h are shown in Figure 8C.

As anticipated, 5-FU was released from the medicated materials in a sustained manner over a longer period of time. However, the sustained release details were significantly different in terms of the initial manner of release and sustained drug release rates. The composite particles S2 released 33.4% ± 4.3% of the loaded 5-FU in the first hour, showing a typical initial burst-release effect. After 6 h, approximately 70.6% ± 4.5% of the loaded 5-FU was freed into the dissolution media. In sharp contrast, the core-shell particles S3 and the particles from the hybrid films S4 released no detectable 5-FU during the first half hour, and only 2.7% ± 0.9% and 3.4% ± 1.1% of the 5-FU were released over the first 2 h, respectively. The blank CA shell layer played an important role in retarding the initial 5-FU release. The release amounts over the first 6 h for S3 and S4 were 30.2% ± 3.9% and 29.8% ± 3.9%, respectively. Later, 5-FU showed sustained release in an almost linear manner up to 30 h. These release profiles of the 5-FU/CA core-shell particles thus ensure the desired asynchronous dual-drug delivery from the hybrid films S4.

**FIGURE 9:**
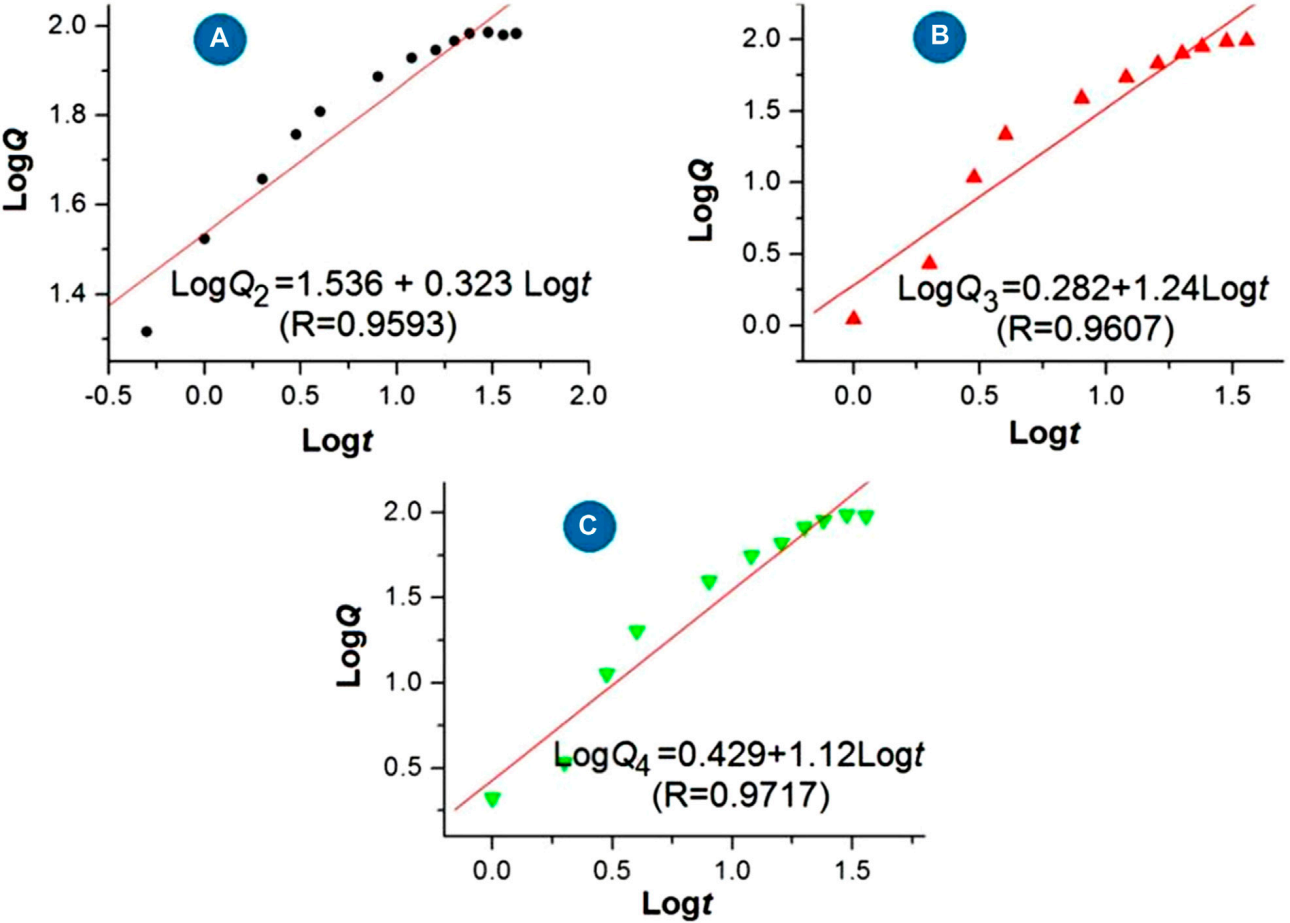
Regression equations for the release of 5-FU from the medicated products: **(A)** homogeneous 5-FU-CA particles S2; **(B)** core-shell 5-FU-CA particles S3; **(C)** core-shell particles released from the hybrid film S4.

The regression equations of the drug 5-FU released from the medicated products are shown in Figure 9 based on the *in vitro* dissolution test data. For the three medicated products, i.e., homogeneous 5-FU/CA particles S2, core-shell 5-FU/CA particles S3, and core-shell particles released from the hybrid films S4, the release equations are LogQ_2_ = 1.536 + 0.323 Logt (R = 0.9593), LogQ_3_ = 0.282 + 1.240 Logt (R = 0.9607), and LogQ_4_ = 0.429 + 1.120 Logt (R = 0.9717), respectively. Based on the critical value of the Peppas equation (Peppas, 1985), the value of the sample S2 (0.323) is less than 0.45, indicating that 5-FU was released from the CA-based particulate composites in a typical Fickian mechanism. Furthermore, the values of the electrosprayed core-shell particles S3 and the particles freed from the hybrid films S4 were n = 1.24 and 1.12, respectively. A value exceeding 0.9 suggests an erosion mechanism based on the Peppas criterion. However, since CA is an insoluble polymer, it is determined that 5-FU was released by a two-step penetration process; the first step involved the penetration of water molecules into the interior of the CA matrix. Later, the second penetration step involved 5-FU molecules passing through the inner channels formed by the water molecules into the bulk dissolution media. This contradiction is unexpected and is attributed to the blank CA coating on the 5-FU/CA composite in the core-shell structures. The Peppas equation is useful for evaluating drug-loaded materials in which the drug molecules are distributed homogeneously throughout the polymeric matrices, but it fails to predict nanomaterials with complicated chamber structures, as has been demonstrated in other investigations (Zhou et al., 2023; Zhao et al., 2024; Zhou et al., 2024a; Wang et al., 2022).

### 3.5 Mechanism of asynchronous dual-drug delivery based on hybrid films

**FIGURE 10:**
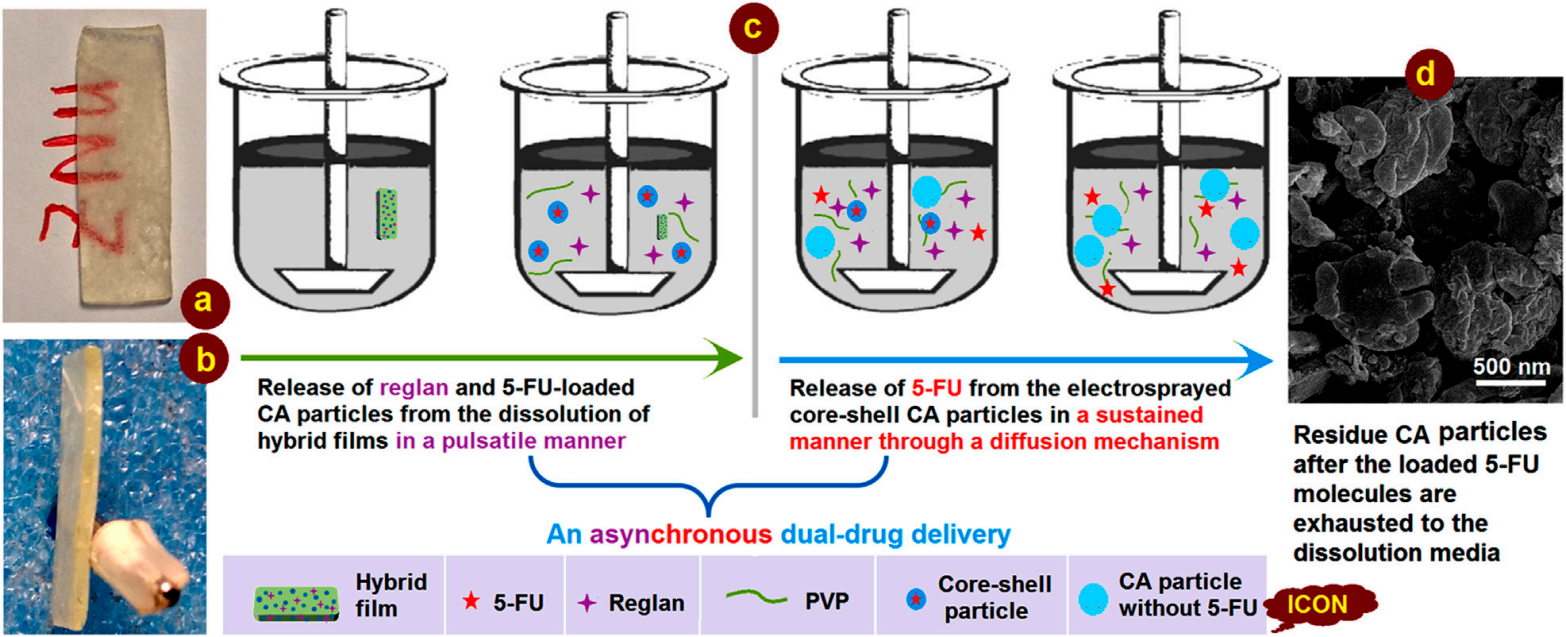
Digital images of the casting films placed in the dissolution vessels: **(A)** surface and **(B)** cross-sectional views; **(C)** mechanism of asynchronous dual-drug delivery; **(D)** SEM image of the core-shell particles after exhaustion of the loaded 5-FU.

This study demonstrates a proof-of-concept strategy for creating synergistic anticancer DDSs based on a combination of the traditional film casting technique and advanced coaxial electrospraying. Knowledge of the working mechanism is generally useful for developing a series of functionally medicated nanomaterials. A diagram and two digital photographs of the casting films are shown in Figure 10. The hybrid film is semitransparent owing to the presence of numerous electrosprayed core-shell particles, as indicated by the red letters “ZNU” in Figure 10A. The film had a thickness of approximately 2.0 mm with a smooth cross-section, reflecting its fragility, which could be improved simply by employing PVP with a higher molecular weight (such as PVP K90) but sacrificing a little of the fast dissolution property.

The asynchronous dual-drug release profiles are shown in detail in Figure 10C. First, the fast dissolution of the soluble PVP film matrix releases the therapeutic ingredient Reglan to achieve the desired antiemetic application in a pulsatile manner. Then, the loaded electrosprayed core-shell particles are simultaneously released into the dissolution media; because of the insoluble CA shell coating on the core drug/polymer medicated composites, there is no initial drug release. Later, the penetration of water molecules into the core-shell particles causes a slight swelling of the particles. In turn, this creates passages for material transportation between the solid particles and the dissolution media for sustained release of the encapsulated 5-FU molecules. Because of the high amount of drug loading in the core section, the CA particles are flattened after 5-FU depletion. This can be deduced from the SEM images of the residue particles (Figure 10D), which were fetched from the dissolution vessels and dried naturally.

## 4 Conclusion

In a pioneering effort, the traditional film casting method was combined with coaxial electrospraying to develop a novel bioengineering strategy for hybrid films. The prepared hybrid films were designed to incorporate two active ingredients, Reglan and 5-FU, with Reglan being homogeneously distributed throughout the polymeric film matrix of PVP K30 while 5-FU was loaded into core-shell particles with a blank CA coating. The coaxial electrospraying process for generating the core-shell particles was optimized and carefully recorded. The SEM and TEM images demonstrated that the casting films were solid and that the loaded particles had distinct core-shell structures. XRD and ATR-FTIR assessments suggested that the two drugs presented in the films were in an amorphous state owing to the favorable secondary interactions between Reglan and PVP and between 5-FU and CA. *In vitro* dissolution tests were used to verify that the desired asynchronous dual-drug delivery could be realized for the potential treatment of colon cancer through the oral administration of the drugs. Both the microformation mechanism of the electrosprayed particles and the asynchronous dual-drug delivery mechanism of the hybrid films are proposed herein.

Effective, safe, and convenient cancer therapy remains one of the greatest concerns in many medical and clinical fields today (Chen et al., 2022; Murugesan and Raman, 2022; Tang et al., 2022; Assi et al., 2023; Wang et al., 2023d; Man et al, 2023; Meng et al., 2022). Numerous active ingredients have been demonstrated for their usefulness through laboratory experiments (Yang et al., 2024a; Duan et al., 2024; Zhang et al., 2024d); meanwhile, new types of pharmaceutical excipients (particularly those based on polymers) are being developed continuously (Lu et al., 2022; Shen et al., 2022; Ali et al., 2023; Yang et al., 2024b; Zhang et al., 2024e; Javadi and Mohsenzadeh, 2024; Riaz et al., 2024), and novel biosourced drug carriers such as exosomes are being considered for DDSs (Agrawal et al., 2024; Guo et al., 2024). Combining the advantages of advanced techniques with those of traditional methods to create novel medical materials from these active ingredients and excipients is an evergreen interdisciplinary challenge for researchers. The protocols reported herein provide a pioneering example for this frontier topic in bioengineering and nanomedicine.

## Data Availability

The original contributions presented in the study are included in the article/supplementary material, and any further inquiries may be directed to the corresponding authors.
