# Corrigendum: Hybrid films loaded with 5-fluorouracil and Reglan for synergistic treatment of colon cancer via asynchronous dual-drug delivery

**DOI:** 10.3389/fbioe.2025.1597014

**Published:** 2025-06-13

**Authors:** Hairong Mao, Jianfeng Zhou, Liang Yan, Shuping Zhang, Deng-Guang Yu

**Affiliations:** ^1^ College of Chemistry and Chemical Engineering, Zhengzhou Normal University, Zhengzhou, Henan, China; ^2^ School of Materials and Chemistry, University of Shanghai for Science and Technology, Shanghai, China; ^3^ School of Optical-Electrical and Computer Engineering, University of Shanghai for Science and Technology, Shanghai, China

**Keywords:** synergistic therapy, hybrid films, colon cancer, asynchronous dual-drug delivery, coaxial electrospraying, casting, tumor-targeted therapy

In the published article, there was an error in Figure 6 as published. The highly similarity of XRD patterns of S2, S3 and CA may mislead the readers. Thus, all the three samples were re- prepared and all the raw polymers and samples were re-tested. The corrected Figure 6 and its caption “FIGURE 6 X-ray diffraction (XRD) patterns of the four starting components (Reglan, 5-FU, PVP, and CA) and their homogeneous composite S2, heterogeneous composite S3, and hybrid casting film S4.” appear below.

**FIGURE 6:**
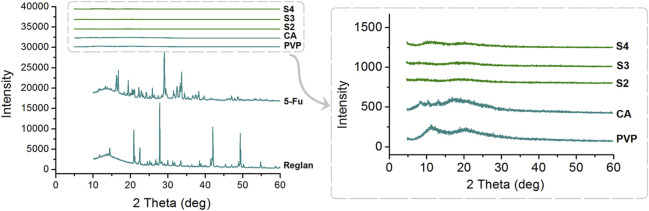
X-ray diffraction (XRD) patterns of the four starting components (Reglan, 5-FU, PVP, and CA) and their homogeneous composite S2, heterogeneous composite S3, and hybrid casting film S4.

The authors apologize for this error and state that this does not change the scientific conclusions of the article in any way. The original article has been updated.

